# Clinicopathological Features and Status of Programmed Death Ligand-1 (PD-L1) Expression in Lung Cancer: A Single Centre Study From North India

**DOI:** 10.7759/cureus.35056

**Published:** 2023-02-16

**Authors:** Firdous Ganie, Nazia Mehfooz, Farhana Siraj, Umar H Khan, Suhail Mantoo, Amrit Dhar, Mohmad Hussain Mir, Rafi A Jan, Sonaullah Shah, Syed Mudasir Qadri

**Affiliations:** 1 Internal and Pulmonary Medicine, Sher-i-Kashmir Institute of Medical Sciences, Srinagar, IND; 2 Internal Medicine, Sher-i-Kashmir Institute of Medical Sciences, Srinagar, IND; 3 Internal and Pulmonary Medicine, Sher-I-Kashmir Institute of Medical Sciences, Srinagar, IND; 4 Medical Oncology, Sher-i-Kashmir Institute of Medical Sciences, Srinagar, IND

**Keywords:** pd-l1 expression, immunohistochemistry, pathology, oncology, lung cancer

## Abstract

Introduction

Programmed death ligand-1 (PD-L1) is an immunological checkpoint that supports the inhibition of the anti-tumor immune system. A higher level of PD-L1 expression was also discovered on the cell surfaces of several cancer cells, including non-small cell lung carcinoma (NSCLC). Identifying individuals who would benefit from PD-1/PD-L1 antibody immunotherapy is crucial in the era of precision medicine. The study's objective was to assess the distribution and degree of PD-L1 ligand expression in various forms of lung cancer and examine its link to clinicopathological variables.

Methods

This prospective, observational, cross-sectional study was done in a tertiary care hospital in North India over 2 years from 2019 to 2021. A total of 100 patients diagnosed with lung cancer through either endobronchial or image-guided biopsies were enrolled. The biopsy specimens of lung cancer patients have been subjected to immunohistochemistry (IHC) staining. PD-L1 expression was positive when at least 1% of tumor cells were stained. In our study, we used the rabbit monoclonal Anti-PD-L1 antibody (CAL10) (ab237726) (Abcam Plc, UK).

Results

Of the 100 patients, Squamous cell carcinoma (SQCC) was the predominant histological pattern. The mean age of the study group was 57.26 ± 10.53 years. High PDL-1 positivity (>50% ) is seen in a total of 10 patients, while low PD-L1 positivity (1-50%) is seen in 24 patients. Of all patients with high PD-L1 positivity (n=10), 80% had stage IV at the time of diagnosis. However, on similar lines, 71 % of patients with low PD-L1 positivity presented with stage IV at the time of diagnosis. (p value=0.09). Among 10 patients with epidermal growth factor receptor (EGFR) positive status, high PD-L1 positivity is seen in 20%. Among 3 patients with anaplastic lymphoma kinase (ALK) positive status, only one patient showed high PD-L1 positivity, whereas negative PDL-1 was seen in 2 patients, which was not statistically significant.

Conclusion

The management of lung cancer is driven by precision medicine, including PDL-1 expression, which correlates with immune checkpoint inhibitor response. In our cohort, PD-L1 expression appears to be mostly linked to the squamous cell subtype of lung cancer, with elevated tumor stage and mediastinal lymphadenopathy in Kashmiri people. Other oncogenic driver mutations are not connected to PD-L1 expression. The function of PD-L1 expression in lung tumors requires more study.

## Introduction

Lung cancer is the second most prevalent cancer diagnosed and one of the top causes of cancer-related fatalities in 2020, accounting for approximately one out of every ten malignancies diagnosed and one out of every five deaths [1]. According to one study, lung cancer's annual crude incidence rate was 4.005 per 100,000 people, and the prevalence was 5.4% in Kashmir Valley, a northern Indian region. The age-adjusted incidence rate was 11.2, with more than three-quarters of the cases occurring in the 40 to 69-year-old age range [2].

Since the 2015 WHO classification, immunohistochemistry (IHC) has played an essential role in lung cancer diagnosis. Aside from the distinction between small and non-small cell carcinoma (NSCLC), patients' treatment preferences are closely related to histologic subtypes of non-small cell carcinoma, as shown by IHC findings. Also, the categorization of lung cancer has improved because of the use of IHC [3]. Programmed death 1 (PD-1) and its ligands, PD-L1 and PD-L2, have emerged as critical inhibitory signaling pathways that govern T-cell response and preserve peripheral tolerance. Programmed death ligand-1 [PD-L1], also known as CD274, is an immunological checkpoint that aids in anti-tumour immune system suppression [4]. The PD-L1 ligand binds to the PD-1 receptor on activated T-cells, resulting in immune system suppression [4,5]. During inflammation, the interaction of PD-1 and PD-L1 prevents an autoimmune response in peripheral tissues [6].

PD-L1 expression was also elevated on the cell surface of various cancer cells, including NSCLC. Cancer cells are thought to avoid the immune response by expressing PD-L1 [4]. Identification and characterization of factors that predict patients who respond well to immunotherapy appear to be critical, particularly in the case of the two major histopathological subgroups of NSCLC, adenocarcinoma and squamous cell carcinoma [7]. Immune checkpoint inhibitors treatment, such as anti-PD 1 (nivolumab, pembrolizumab, cemiplimab) or anti-PD-L1 ( atezolizumab, durvalumab) or anti-CTLA-4 (ipilimumab), has recently been emphasized in several NSCLC clinical trials. In the era of precision medicine, it is imperative to screen patients who are most likely to benefit from PD-1/PD-L1 antibody immunotherapy [7,8]. PD- L1 expression has been studied in several prior investigations. However, the PD-L1 positive criterion in SCLC was arbitrary, and the antibodies utilized differed. [9] Additionally, the association of PD-L1 expression with clinicopathological factors is unclear.

The study aimed to determine the level of expression and localization of PD-L1 ligands in various lung cancers and analyze the relationship between PD-L1 expression and clinicopathological factors to determine its prognostic and predictive value.

## Materials and methods

This was a prospective, observational, cross-sectional study done in 1200 bedded tertiary care university teaching hospital in North India. The study was conducted over 2 years, from 2019 to 2021. The Institutional Ethics Committee, Sher-i-Kashmir Institute of Medical Sciences, Srinagar, India, approved the study (approval number: #74/2021), and all procedures followed the Helsinki Declaration. After receiving written informed consent, the participants were enrolled. 

A total of 100 patients diagnosed with lung cancer through either endobronchial or image-guided biopsies were enrolled. A detailed history regarding presenting complaints, family history, smoking status, co-morbidities, and demographic data were collected, and a general physical examination was done. Relevant investigations were done for staging the disease. Disease staging was done according to AJCC-IASLC-UICC (American Joint committee for Cancer Control- International Association for Study of Lung Cancer- Union for International Cancer Control)-8th edition [10].

Determination of PD-L1 expression by IHC staining

The biopsy specimens of lung cancer patients were subjected to IHC staining. For assessing the expression of PD-L1, a tumor proportion score was used, as shown in Table 1. The percentage of viable tumor cells that exhibit partial or complete membrane staining at any intensity was used to determine the expression level of PD-L1. When at least 1% of tumor cells were stained, PD-L1 expression was found to be positive [11]. In our study, we used the rabbit monoclonal Anti-PD-L1 antibody [CAL10] (ab237726) (Abcam Plc, UK). IHC staining was performed manually according to the instructions provided in the manual attached to the primary antibody. Upon cellular localization, membranous staining was taken as positive.

**Table 1:** Tumour Proportion score determined according to the percentage of cells stained.

SCORE	LOCALIZATION	CELLS STAINED (%)
NEGATIVE	MEMBRANOUS	< 1
LOW POSITIVE	MEMBRANOUS	1-49
HIGH POSITIVE	MEMBRANOUS	> 50

Statistical Analysis

The continuous variable was summarised as mean, standard deviation (SD), or median and interquartile range (IQR) appropriate for descriptive statistics. The categorical variables are described as frequencies and percentages. All data were assessed for normality. Student's t-test, Fisher's exact test, Pearson Chi-square test, and Mann-Whitney U test were used wherever applicable. Statistical analysis was done using IBM SPSS Statistics for Windows, Version 28.0 (Released 2021; IBM Corp., Armonk, New York, United States). The significance level was set at p < 0.05.

## Results

Of the 100 subjects, there was a male predominance (n =79). Out of this, squamous cell carcinoma (SQCC) was the predominant histological pattern found in 51 % of male patients, while adenocarcinoma (ADC) was the main histological pattern in females. The mean age of the study group was 57.26 ± 10.53 years. Out of 100 patients, 78 were smokers, and 22 were non-smokers, with SQCC being the predominant histopathological pattern (HPE) found in smokers and ADC being the predominant HPE in non-smokers. Cough was the predominant symptom found in 57% of patients. 52% were diagnosed with stage IV disease, 8 with stage IIIC, 12 with stage IIIB, 18 with stage IIIA, 9 with stage IIB, and only one with stage IIA. On Contrast-enhanced computed tomography (CECT) of the chest, hyper-enhancing mass was mainly found in 86 % of patients. Table 2. depicts the clinicopathological features of the subjects.

**Table 2:** Clinicopathological features of the subjects. SD: standard deviation; PD-L1: programmed death ligand 1; CECT: contrast-enhanced computed tomography.

Variable	N	High Positive (PD-L1: >50%)	Low Positive (PD-L1: 1-50%)	Negative (PD-L1: <1%)	p-value
Total patients	100	10	31	59	N/A
Gender (Male)	79	9	24	46	0.66
Age, years (mean ±SD)	57.26 ±7.8	53.90 ± 10	60 ± 11	56 ± 10	0.17
Active Smoker	78	9	24	45	0.623
Histology					
Squamous cell carcinoma	55	8	21	26	0.01
Adenocarcinoma	29	1	7	21	
Adenosquamous	3	1	2	0	
Small cell cancer	13	0	1	12	
Staging					
IIA	1	0	0	1	0.002
IIB	9	0	1	8	
IIIA	18	1	5	12	
IIIB	12	0	1	11	
IIIC	8	1	2	5	
IV	52	8	22	22	
Symptoms					
Cough	57	8	15	34	0.212
Shortness of breath	45	3	15	27	0.587
Haemoptysis	38	4	11	23	0.940
Chest pain	32	1	8	23	0.129
Hoarseness of voice	5	2	1	2	0.07
CECT Chest findings					
Hyperenhancing mass	86	9	24	53	0.20
Hilar enlargement	25	2	5	18	0.30
Lymphadenopathy	64	9	26	29	0.001
Consolidation/collapse	25	1	12	12	0.08
Pulmonary nodules	21	0	8	13	0.20

Association between PD-L1 and clinicopathological features

High PDL-1 positivity (>50% ) is seen in 10 patients, of which 90% were males, and 10% were females. Low PD-L1 positivity( 1-50%) is seen in 24 patients, and it is also higher in males constituting 77.4% in males and 22.6 % in females. PD-L1 negativity was also more in males than females. The mean age of lung cancer is 57.26 ± 10.53, with high positivity of PD-L1 found in 10 patients with a mean age of 53.90 ± 7.8, low positivity in 31 patients with a mean age of 60.00 ± 11.65, and negative PD-L1 in 59 patients with mean age 56 ± 10) (p-value- 0.17). Chronic cough was the predominant symptom in patients with lung cancer. Among these, strong PD-L1 positivity was seen in 80% of patients presenting with a cough. Hemoptysis was present in 38 patients. Out of 10 cases with strong PD-L1 positivity, hemoptysis was found in 40% of patients. None of the clinical features was found to have a statistically significant correlation with the PDL1 expression in the studied population.

Correlation of PD-L1 expressions in lung cancer subtypes.

Out of 10 cases of high PD-L1 positivity( >50%), the majority were (80%) squamous cell carcinoma. Low PD-L1 positivity is more in SQCC, constituting 67.7%. Besides, negative PDL-1 status (<1%) was also found in 44% of SQCC, 35.6% of ADC, and 20% of SCC (p=0.011). On further comparison of the PD-L1 status of SQCC with other HPE types, it was statistically significant (p =0.02).

Correlation of PD-L1 with a staging of lung cancer

Of all patients with high PD-L1 positivity (n=10), 80% had stage IV at the time of diagnosis. However, on similar lines, 71% of patients with low PD-L1 positivity presented with stage IV at the time of diagnosis (p value=0.09). Comparison of the PD-L1 status of Stage IV with other stages was statistically significant (p = 0.002).

Correlation of Programmed Death Ligand (PD-L1) Expression with chest imaging (CECT- chest)

Out of 86 patients with a hyper-enhancing lesion on CECT, high PD-L1 positivity status was seen in 10% of patients, low PD-L1 positivity in 28%, and absent PD-L1 status in 61% of patients. Lymphadenopathy was found in 64% of patients and absented in 36% of patients.

Correlation of PD-L1 with EGFR and ALK mutation

Molecular analysis of markers was done in 40 patients, out of which epidermal growth factor receptor [EGFR] was positive in 10 patients and absent in 30 patients. Anaplastic lymphoma kinase (ALK) was analyzed in 44 patients, ALK-positive in 3 patients, and negative in 41 patients. Table 3. depicts the correlation of PDL-1 Status with EGFR and ALK. Among 10 patients with EGFR-positive status, high PD-L1 positivity is seen in 20%. However, there was no statistical significance (p = 0.07). Among 3 patients with ALK-positive status, only one showed high PD-L1 positivity, whereas negative PDL-1 was seen in 2 patients (p = 0.38). 

**Table 3:** Correlation of PDL-1 Status with EGFR and ALK. PD-L1: programmed death ligand 1; EGFR: epidermal growth factor receptor; ALK: anaplastic lymphoma kinase

Variable	N	High Positive (PD-L1: >50%)	Low Positive (PD-L1: 1-50%)	Negative (PD-L1: <1%)	p-value
EGFR	10	2	0	8	0.07
ALK	3	1	0	2	0.38

## Discussion

The second most common cancer among Kashmiris is lung cancer [12]. PD-L1 expression, among other markers, has been linked to a poor prognosis in lung cancer. This hospital-based study aimed to find out how common PD-L1 expression is and to look at the relationship between clinicopathological features and PD-L1 expression. PD-L1 expression results obtained using different assays are incompatible because different pharmaceutical manufacturers use and test PD-L1 antibodies from different manufacturers on different platforms. Our study used Rabbit Monoclonal Antibody Clone (CAL 10) isotype IgG.

Our study looked at 100 lung cancer cases with either image-guided or fibro-optic bronchoscopy (FOB) guided endobronchial biopsy. Our study's PD-L1 positivity (41%) was comparable to that of Pawelczyk et al. [13], which found positive PD-L1 expression in 32.5% of NSCLC patients. Farrag et al. discovered that PD-L1 is over-expressed in lung cancer, particularly in NSCLCs, where 71% of positive cases showed high positive expression and 59.1% of positive cases showed low positive expression [14]. The varied expression of PD-L1 in lung cancer may necessitate adopting several evaluation criteria and scoring systems. The tumor percentage score (TPS) [ratio of PD-L1 stained tumor cells to the total viable tumor cells], which is presently often employed in diagnostics, was used in our investigation. Multiple antibody clones may cause variations in the percentage of patients expressing PD-L1 in NSCLC cells in various studies (22C3, 22-8, SP142, and SP263). Clinical studies validated these antibodies for several PD-1/PD-L1 inhibitors [15].

Smoking is considered one of the primary risk factors for lung cancer [16]. Among our group, it was mostly associated with cigarette smoking in men, hookah smoking in women, and environmental smoking. Our study indicated a substantial link between smoking and lung cancer, with men making up most of those affected. Although we did not statistically calculate the risk of lung cancer related to the quantity and frequency of cigarettes smoked in our investigation, many other studies have shown that the length of smoking would have a bigger impact than the quantity smoked daily [2,15]. Several research studies indicate that smoking can enhance PD-L1 expression in people with lung cancer. This may be caused by cigarette carcinogens, which alter tumors' development, resulting in a novel antigen and a rise in PD-L1 expression. Smoking can also promote inflammation, which includes the activation of T cells and the release of inflammatory cytokines like interferon-gamma, as well as increased PD-L1 expression [17]. Our research, however, did not discover any evidence of a connection between smoking and PD-L1 expression. When we looked for a relationship between the expression rate of PD-L1 and any clinical characteristics, we discovered that there was none.

As the predominant histological type in our analysis was SQCC, we discovered that PD-L1 expression was much higher in this type (p=0.02) than in the adenocarcinoma pattern, as shown in a related study by Cooper et al. [18]. Additionally, Boothman et al. demonstrated that squamous cell cancer expressed more PD-L1 than non-squamous carcinoma [19]. When PD-L1 expression was compared to lung cancer staging, stage IV was found to have a higher expression rate, with 8% of cases showing strong positive and 22% showing low positivity. Thus, we may conclude that PD-L1 expression would be more intense at the higher cancer stage. This was comparable to research that discovered a significant difference between stage I adenocarcinoma tumors and higher infiltration stages in terms of decreased PD-L1 expression rates [20]. According to Lin et al. [11], PD-L1 expression was associated with increased aggressiveness at a later stage in NSCLC.

Except for lymphadenopathy, there was no correlation between PD-L1 expression and CECT chest results in our investigation. Similarly, it was discovered in certain studies that lymphovascular invasion was connected to PD-L1 expression, which was more intense, and those patients appeared to benefit more from immunotherapy [21,22]. Additionally, we attempted to determine if the expression of PD-L1 was significantly correlated with the presence of the critical oncogene driver mutations EGFR and ALK rearrangements. In a study by Yang et al. [23], it was discovered that there was no correlation between PD-L1 expression and driver mutations, such as EGFR and ALK. However, Zhang et al. [20] discovered a correlation between PD-L1 and EGFR but not ALK, and Sliva et al. discovered a correlation between PD-L1 and ALK [24]. This discovery suggests that patients with various tissue types and driver gene mutations may respond to ICI therapy differently, although this has to be proven in more research.

Our study had a few limitations; first, the fact that our study was a single-center study with a small sample size of 100 made it challenging to draw inferences. Second, whereas squamous cell carcinoma predominated in our study, the national trend is increasing toward adenocarcinoma as the prevalent histological type [25], which may make it challenging to extrapolate our findings.

## Conclusions

The management of lung cancer is driven by precision medicine, including PDL-1 expression, which correlates with ICI response. In our cohort, PD-L1 expression appears to be mostly linked to the squamous cell subtype of lung cancer, with elevated tumor stage and mediastinal lymphadenopathy in Kashmiri people. Other oncogenic driver mutations are not connected to PD-L1 expression. The function of PD-L1 expression in lung tumors requires more study.
